# Opportunities for Savings in Risk Arrangements for Oncologic Care

**DOI:** 10.1001/jamahealthforum.2023.3124

**Published:** 2023-09-15

**Authors:** Bruce E. Landon, Miranda B. Lam, Mary Beth Landrum, J. Michael McWilliams, Laurie Meneades, Alexi A. Wright, Nancy L. Keating

**Affiliations:** 1 Department of Health Care Policy, Harvard Medical School, Boston, Massachusetts; 2 Division of General Medicine, Beth Israel Deaconess Medical Center, Boston, Massachusetts; 3 Department of Radiation Oncology, Brigham and Women’s Hospital, Dana-Farber Cancer Institute, Boston, Massachusetts; 4 Division of General Internal Medicine, Brigham and Women’s Hospital, Boston, Massachusetts; 5 Department of Medical Oncology, Dana-Farber Cancer Institute, Boston, Massachusetts; 6 Center for Psycho-Oncology and Palliative Care Research, Dana-Farber Cancer Institute, Boston, Massachusetts

## Abstract

**Importance:**

As the US accelerates adoption of alternative payment through global payment models such as Accountable Care Organizations (ACOs) or Medicare Advantage (MA), high spending for cancer care is a potential target for savings.

**Objective:**

To quantify the extent to which ACOs and other risk-bearing organizations operating in a specific geographic area (hospital referral region [HRR]) could achieve savings by steering patients to efficient medical oncology practices.

**Design, Setting, and Participants:**

This observational study included serial cross-sections of Medicare beneficiaries with cancer from 2010 to 2018. Data were analyzed from August 2021 to March 2023.

**Main Outcomes and Measures:**

Total spending and spending by category in the 1-year period following an index visit for a patient with newly diagnosed (incident) or poor-prognosis cancer.

**Results:**

The incident cohort included 1 309 825 patients with a mean age of 74.0 years; the most common cancer types were breast (21.4%), lung (16.7%), and colorectal cancer (10.0%). The poor prognosis cohort included 1 429 973 (mean age, 72.7 years); the most common cancer types were lung (26.6%), lymphoma (11.7%), and leukemia (7.3%). Options for steering varied across markets; the top quartile market had 10 or more oncology practices, but the bottom quartile had 3 or fewer oncology practices. Total spending (including Medicare Part D) in the incident cohort increased from a mean of $57 314 in 2009 to 2010 to $66 028 in 2016 to 2017. Within markets, total spending for practices in the highest spending quartile was 19% higher than in the lowest quartile. Hospital spending was the single largest component of spending in both time periods ($20 390 and $19 718, respectively) followed by Part B (infused) chemotherapy ($8022 and $11 699). Correlations in practice-level spending between the first-year (2009) and second-year (2010) spending were high (>0.90 in all categories with most >0.98), but these attenuated over time.

**Conclusions and Relevance:**

These results suggest there may be opportunities for ACOs and other risk-bearing organizations to select or drive referrals to lower-spending oncology practices in many local markets.

## Key Points

**Question:**

What are the opportunities for accountable care organizations (ACOs) and other risk-bearing organizations operating in a specific geographic area to achieve savings by steering patients to efficient medical oncology practices in a region?

**Findings:**

This cohort study of 2 739 798 patients found that opportunities for steering varied across hospital referral regions (HRRs); the top quartile HRR in number of practices had 10 or more practices, but the bottom quartile had 3 or fewer oncology practices. Overall, total spending in the highest spending quartile of practices in a market was 19% higher than in the lowest quartile, and practice-level spending tendencies were persistent over time.

**Meaning:**

These results suggest that there may be opportunities for ACOs and other risk-bearing organizations (eg, capitated health plans) to select or drive referrals to lower-spending oncology practices in many local markets.

## Teaser

This cohort study examines opportunities for accountable care organizations and other risk-bearing organizations to achieve savings by steering patients to efficient medical oncology practices operating in specific geographic areas.

## Introduction

An estimated 1.9 million people in the US were diagnosed with cancer in 2022.^1^ Spending on cancer care is high and growing rapidly, from an estimated $162 billion in 2015 to a projected $221 billion in 2030.^2^ The growth in spending on cancer care is attributed to the aging of the population and improvements in survival for many types of cancer,^2^ in part due to effective but high-priced treatments.^1,3^ However, many newer treatments offer only marginal improvements in life expectancy, which sometimes are accompanied by substantial negative effects on quality of life.^4,5,6^

As the US expands alternative payment through global payment models such as accountable care organizations (ACOs) or Medicare Advantage (MA), high spending for cancer care is a potential target for savings. In 2016, the Centers for Medicare & Medicaid Services (CMS) initiated the Oncology Care Model,^7^ an alternative payment model for oncology practices treating patients with chemotherapy; this model will be followed by the Enhancing Oncology Model beginning in 2023.^8^ Previously, CMS proposed a Radiation Oncology Model, but it has been indefinitely delayed.^9^

Several prior analyses have found substantial variability in spending on care for patients with cancer across geographic regions, with differences primarily in the use of chemotherapy and hospitals.^10,11,12,13^ These studies suggest there may be opportunities for savings under global payment due to improved care management, including potential savings related to selectively steering patients to low-spending oncology practices whether through selective contracting or other mechanisms. Organizations accepting risk for the costs of care such as an ACO or MA plan operate in specific geographic areas; however, the relevant unit to identify high-performing groups is within these geographic markets rather than across them.

We used data on the treatment of patients with cancer for beneficiaries enrolled in fee-for-service Medicare to examine the spending patterns of oncology practices operating in regional health care markets where patients could conceivably obtain specialty care from any clinician in the market.^14^ Opportunities to save from steering patients to specific practices are stronger if there is greater variation in spending, it persists over time, and is consistent across categories of spending where variation is more likely to reflect variation in practice patterns as opposed to selection. We first sought to quantify the extent to which risk-bearing organizations could achieve savings by selecting efficient clinical practices in that market. In addition, we sought to examine the extent to which oncology practices’ relative spending was consistent over time. As a secondary outcome, we examined variation in subcategories of cancer spending in 2 different ways to gauge potential savings. First, we estimated variation in spending in dollars, which we expected to be higher when mean spending was higher and when practice patterns varied. Second, we calculated coefficients of variation to identify categories of spending in which spending varied more relative to its mean. The latter may suggest priority areas to focus on for oncologists and practice managers seeking to improve the value of cancer care in the context of local health care practice patterns.

## Methods

### Data and Study Populations

This project was approved by the CMS Privacy Board and the Harvard Medical School institutional review committee, which also waived the requirement for obtaining informed consent because the study was deemed minimal risk and used existing available data not collected for this study. Using 100% Medicare data, we identified traditional fee-for-service Medicare beneficiaries between 2009 and 2017 diagnosed with any type of cancer, excluding nonmelanoma skin cancers. We restricted to those who also had Part D prescription drug coverage (ranging from 50% in 2009 to 70% in 2018), which was necessary to identify oral chemotherapy. Cases were identified based on having at least 1 inpatient visit with a primary diagnosis or 2 face-to-face outpatient visits (≥30 days apart) with an *International Classification of Diseases, Ninth Revision (ICD-9)* or *ICD-10* diagnosis code for cancer (see eAppendix in Supplement 1 for details). We focused our assessment of spending on the first year after the index date (first claim identifying diagnosis or new recurrence), which is the time when diagnostic and staging evaluations are completed and treatments are initiated.

From these cases, we identified 2 study cohorts. The incident cohort included all patients with a new diagnosis of cancer during a study year (2009-2017) who were continuously enrolled in Medicare Part A and Part B with no Medicare Advantage enrollment in the 2-year period before their first cancer claim and had no cancer-related claims over that period. We used a 2-year look back because some patients with previously treated cancers (eg, prostate, breast) were missed with a 1-year lookback whereas few additional cases were identified with a 3-year look back. Nonetheless, this might include a small percentage of patients with recurrent or progressive cancers for which they did not receive any cancer-related care in the 2 prior years. Of note, to avoid excluding the youngest Medicare-eligible patients, we included newly enrolled in Medicare with only a 1-year lookback. We excluded this latter group of patients in sensitivity analyses and found minimal differences.

The poor-prognosis cohort was identified based on an algorithm developed by Obermeyer et al^15^ and updated by our team^16^ that included patients with poor-prognosis primary diagnoses, metastatic or ill-defined malignant diseases, and hematologic malignant diseases designated as relapsed or not in remission. As with the incident cohort, we identified a prospective cohort from the time of their first relevant code and then followed patients for the 1-year period after diagnosis or until death (within 1 year). For the poor-prognosis cohort, we required 1 year of continuous enrollment before the first qualifying claim.

Oncology practices were identified as all tax identification numbers (TINs) that included at least 1 office visit claim with a CMS specialty code of medical oncology, hematology/oncology, hematology, or gynecologic oncology. Patient care episodes were attributed to an oncology practice TIN based on the plurality of evaluation and management visits with a cancer diagnosis code to an oncology practice as defined herein during the 1-year period of care following the qualifying first claim.^17^ Oncology practices could range from small single-specialty oncology practices to larger multispecialty practices, some of which included radiation oncologists and/or surgeons who also care for patients with cancer. We divided the small number of practices spanning multiple hospital referral regions (HRRs) into practice/HRR subunits and assigned patients accordingly. Approximately 52.1% of the incident cohort (ranging from 71.5% for breast cancer to 9.6% for prostate cancer) and 66.3% of the poor-prognosis cohort (ranging from 88.3% for breast cancer to 65.0% for lung cancer) received care from an oncologist in the year following diagnosis.

We restricted these analyses to patients assigned to oncology practices with at least 20 assigned cancer cases (including both the incident and poor-prognosis cohorts) in at least 1 of the study years.

### Outcomes

The main outcome of interest was total spending in the 1-year period following the index diagnosis date. We chose 1 year because that is the spending period used for ACO spending calculations. Because prices in Medicare are set administratively, total spending can be thought of as a summary measure of the quantity and mix of services delivered. We summed Medicare payments, coinsurance amounts, and payments from other primary payers to assess total medical spending on all services covered by Medicare Parts A, B, and D. We assigned every claim to 1 of 13 service subcategories following the definitions of Keating et al,^7^ who designed the categories to be mutually exclusive and cancer relevant. Though much of this spending is not directly with the oncology practice, our approach assumes that oncologists generally have the most direct control over medical decisions and consequent spending in the year after a new cancer diagnosis.

### Geographic Regions

We used the HRRs defined in the Dartmouth Atlas of Health Care for our geographic regions/markets in this study. Patients were assigned to an HRR based on their zip code of residence. The HRRs were defined by the Dartmouth Atlas of Health Care to reflect regional health care markets based on referral patterns for tertiary care.^18,19^

### Statistical Analyses

For each calendar year for each cohort, we estimated linear regression models that included random effects for each practice and fixed effects for HRR. Thus, the model estimates practice variation within a market while also negating the effects of variation in prices across geographic areas. We included control variables for cancer type as categorized by the Oncology Care Model as well as patient sociodemographic characteristics (age defined in 5-year intervals, documented sex, race and ethnicity based on the RTI race variable included in the Medicare data [defined as Black (non-Hispanic), Hispanic, White (non-Hispanic), or other (including Asian/Pacific Islander, American Indian/Alaska Native, and unknown^20^)]), and comorbid conditions from the Chronic Conditions Warehouse (CCW) as of the index year. We identified patients with low incomes and limited financial resources based on enrollment in Medicaid or the Part D Low-Income Subsidy, and classified patients into 1 of 3 mutually exclusive groups: enrolled in full Medicaid for at least 1 month, enrolled in partial Medicaid for at least 1 month (without full Medicaid), or enrolled in the Part D Low-Income Subsidy for at least 1 month (without any Medicaid coverage). We also included hierarchical condition category risk scores calculated from the claims over the 12 months preceding the index cancer diagnosis.^21,22^ To examine variation in patients’ characteristics across practices that may contribute to spending differences, we also estimated similar models with each control variable as the dependent variable to estimate the extent of variation in patient characteristics across TINs.

For spending overall and by category, we reported the standard deviation (SD) of the within-market variance (in dollars) estimated by the models. We then calculate the standardized coefficient of variation (ratio of the SD to the mean) for each category to describe how variation differed across categories after accounting for differences in mean spending. Finally, we characterized variation in relative terms (overall and by category) by dividing the mean of the top quartile of practices ranked by their adjusted spending estimated from the random effects model by the mean of the bottom quartile. We compared these results for the beginning and end of the study period, defined as the first 2 years (2009-2010) and last 2 years of the study period (2016-2017).

To examine correlations of spending for individual practices over time, we estimated joint models that yielded estimates that were substantially less biased and higher in magnitude than naive estimators (eg, Pearson correlations) that postprocess the estimates obtained from individual models.^23^ Data for this study were analyzed from August 2021 to March 2023. This study used SAS Enterprise Guide statistical software (version 7.15; SAS Institute, Inc) for analyses. This study followed the Strengthening the Reporting of Observational Studies in Epidemiology (STROBE) reporting guidelines for reporting cohort studies (eAppendix in Supplement 1).

## Results

The study cohorts included 1 309 825 patients in the incident cohort and 1 429 973 patients in the poor-prognosis cohort. The mean ages in the incident and poor-prognosis cohorts were 74.0 and 72.7 years, respectively, and approximately 80% of patients were White (Table 1). The most common cancers in the incident cohort were breast (21.4%), lung (16.7%), and colorectal cancer (10.0%), and the most common in the poor-prognosis cohort were lung (26.6%), lymphoma (11.7%), and leukemia (7.3%). To provide an indication of the extent to which patients sort among TINs, adjusted TIN-level SDs of characteristics are presented in eTable 2 in Supplement 1. The highest SDs were for socioeconomic characteristics such as race and ethnicity (11% for Black and 14% for White in the 2009-2010 incident cohort) and Medicaid eligibility (13% for full and 14% for the low-income subsidy), suggesting that some practices served different populations of patients, though analyses that adjusted for these patient characteristics yielded similar results to those that did not. There was minimal variation in the types of cancers seen.

**Table 1.** Characteristics of Study Population by Cohort

Characteristic	Patients, %	
	Incident (n = 1 309 825, 47.8%)	Poor prognosis (n = 1 429 973, 52.2%)
Age, mean, y	74.0	72.7
<65	10.9	13.7
65-69	19.9	24.6
70-74	23.9	20.6
75-79	19.7	17.5
80-84	14.3	13.1
≥85	11.3	10.5
Sex		
Female	62.8	52.5
Male	37.2	47.5
Race and ethnicity		
Black	8.7	9.2
Hispanic	4.6	5.1
White	83.2	81.7
Other^a^	3.5	4.0
Medicaid status		
Full	20.8	21.9
Partial	8.8	8.9
Low-income subsidy	31.9	33.1
Cancer type		
Breast	21.4	6.3
Lung	16.7	26.6
Colon, rectal, or intestinal	10.0	5.5
Lymphoma	7.2	11.7
Leukemia	4.1	7.3
Pancreatic	3.4	5.4
Prostate	3.0	4.2
Other	34.2	33.0

^a^ Other includes American Indian/Alaska Native, Asian/Pacific Islander, and Unknown.

The total number of oncology practices with at least 20 cases in any year decreased from 2394 in 2009 to 2036 in 2017. The mean number of cases per practice increased from 87 in 2009 to 137 in 2017. The mean number of practices per HRR decreased from 8.0 to 6.8 across the study period, but the median number of practices per HRR remained stable at 5. Opportunities for steering patients to the most efficient practices varied across HRRs; the top quartile of HRRs by number of practices had 10 or more oncology practices, but the bottom quartile had 3 or fewer.

### Spending

#### Incident Cohort

Total spending per patient increased from a mean of $57 314 in the first 2 years (2009-2010 cohorts) to $66 028 in the last 2 years (2016-2017 cohorts) (Table 2). Hospital spending was the single largest component of spending ($20 390 and $19 718, respectively) followed by Part B (infused) chemotherapy ($8022 and $11 699). Part D drug spending, which includes oral chemotherapy, increased from $4272 to $6758.

**Table 2.** Spending and Variation in the Incident Cohort (2009/2010 and 2016/2017)

Variable	2009/2010, $					2016/2017, $				
	Mean	Median	Q1 Mean	Q4 Mean	Q4/Q1	Mean	Median	Q1 Mean	Q4 Mean	Q4/Q1
Acute hospital	20 390	20 168	17 710	23 463	1.32	19 718	19 569	16 881	22 919	1.36
Part B chemotherapy	8022	7840	6130	10 240	1.67	11 699	11 543	9611	14 087	1.47
Outpatient procedures	4527	4504	4068	5025	1.24	6153	6131	5705	6650	1.17
Part D	4272	4192	3587	5100	1.42	6758	6714	6060	7561	1.25
Radiation therapy	4054	3995	3200	5025	1.57	4459	4426	3744	5250	1.40
Postacute facility	3226	3197	2662	3842	1.44	2947	2922	2483	3452	1.39
Imaging	2532	2503	2084	3033	1.46	2593	2576	2208	3022	1.37
Outpatient physician	2260	2243	1931	2625	1.36	3063	3039	2692	3486	1.29
Part B nonchemotherapy medications	1973	1833	1045	3143	3.01	2734	2584	1496	4264	2.85
Diagnostic testing	1726	1710	1458	2032	1.39	1705	1681	1415	2041	1.44
Home health	1495	1485	1328	1679	1.26	1374	1367	1226	1533	1.25
Hospice	1387	1380	1236	1552	1.26	1308	1300	1169	1461	1.25
Other Part B services	739	718	541	978	1.81	997	969	724	1325	1.83
DME	713	711	673	757	1.12	520	518	485	560	1.15
Total	57 314	57 080	52 567	62 498	1.19	66 028	65 832	61 393	71 036	1.16

Abbreviation: DME, durable medical equipment.

Spending on nonchemotherapy Part B medications exhibited the most relative within-region variation in the initial 2 years with 3-fold more spending on Part B medications in the most expensive quartile of practices compared with the least (Table 2). Overall, total spending in the highest quartile of practices was 19% higher than in the lowest quartile. In the most recent 2 years, spending in the highest quartile of practices was 16% higher than in the lowest quartile.

#### Poor-Prognosis Cohort

Spending for the poor-prognosis cohort followed a similar pattern (Table 3). Overall, total spending in the highest quartile of practices was 22% higher, which moderated to 17% higher in the last 2 years.

**Table 3.** Spending and Variation in the Poor-Prognosis Cohort (2009/2010 and 2016/2017)

Variable	2009/2010, $					2016/2017, $				
	Mean	Median	Q1 Mean	Q4 Mean	Q4/Q1	Mean	Median	Q1 Mean	Q4 Mean	Q4/Q1
Acute hospital	22 637	22 432	19 380	26 312	1.36	21 729	21 533	18 635	25 204	1.35
Part B chemotherapy	9421	9191	7062	12 233	1.73	13 509	13 341	11 024	16 312	1.48
Outpatient procedures	3502	3488	3047	3991	1.31	4668	4648	4239	5142	1.21
Part D	3872	3780	3010	4906	1.63	8617	8529	7554	9837	1.30
Radiation therapy	2660	2613	2122	3294	1.55	2714	2680	2274	3216	1.41
Postacute facility	3319	3263	2543	4202	1.65	2972	2933	2377	3638	1.53
Imaging	2542	2517	2104	3022	1.44	2527	2510	2136	2955	1.38
Outpatient physician	2035	2023	1711	2390	1.40	2763	2741	2398	3169	1.32
Part B nonchemotherapy medications	2200	2025	1190	3509	2.95	2906	2737	1622	4497	2.77
Diagnostic testing	1412	1391	1150	1716	1.49	1282	1260	992	1618	1.63
Home health	1369	1361	1216	1535	1.26	1246	1242	1115	1389	1.25
Hospice	2155	2136	1880	2459	1.31	1905	1889	1659	2177	1.31
Other Part B services	800	778	589	1053	1.79	1020	989	748	1346	1.80
DME	672	669	629	721	1.15	499	496	461	541	1.17
Total	58 596	58 481	52 929	64 571	1.22	68 357	68 210	63 284	73 735	1.17

Abbreviation: DME, durable medical equipment.

### Spending Variation

Among areas of spending accounting for at least 5% of spending, acute hospital care and infused chemotherapy demonstrated the combinations of the largest absolute variation in spending alongside a high coefficient of variation, suggesting the potentially largest areas for savings. In addition, radiation therapy, Part D spending, and postacute facility costs also exhibited high variation relative to its spending (Figure, focusing on the top 4-6 rows depending on the cohort). These findings remained relatively consistent at the end of the study period. The coefficient of variation for each service category for the poor-prognosis cohort were similar to the incident cohort.

**Figure.**
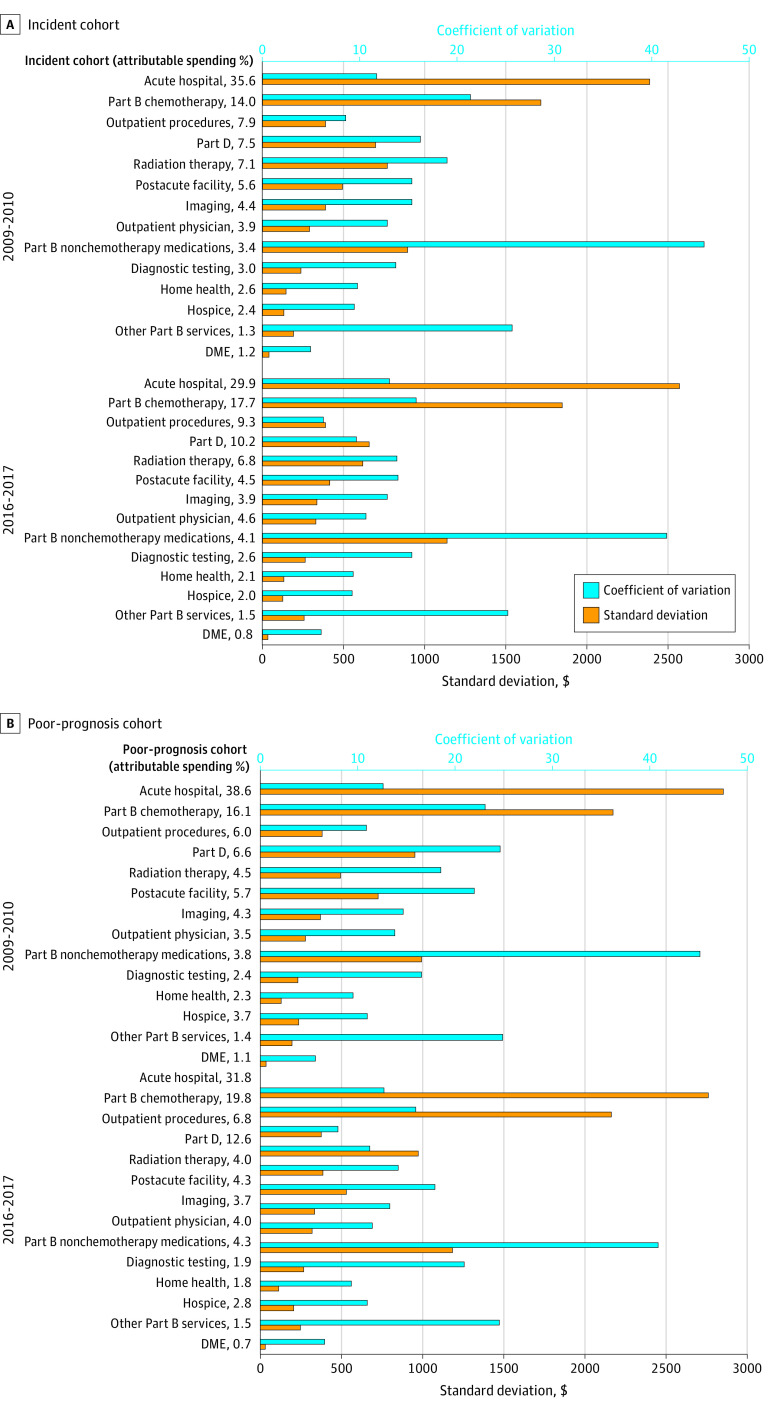
Attributable Spending and Coefficient of Variation in the Incident and Poor-Prognosis Cohorts DME indicates durable medical equipment. Attributable spending is the percent of total spending within a market in the particular category, and the coefficient of variation (standard deviation/mean) shows how much spending in this category varies compared with its share of spending.

### Consistency Over Time and Correlations Between Service Categories

In joint models that accounted for measurement error, correlations in practice-level spending between the first-year (2009) and second-year (2010) spending were high (>0.90 in all categories, with most being >0.98) (Table 4). When examining adjusted correlations between spending in the first and last years (8 years later), correlations were attenuated, but were 0.7 or greater in more than half of the categories, including acute hospital care and chemotherapy. Results were similar in the poor-prognosis cohorts.

**Table 4.** Correlations Between Spending in Year 1 and Year 2, Year 3, and Year 9

Correlation of quartile in 2009	2010	2011	2017
**Incident cohort**			
Acute hospital	0.95	0.97	0.77
Postacute facility	0.95	1.00	0.86
Hospice	1.00	0.74	0.72
Home health	0.90	0.85	0.54
DME	1.00	1.00	0.67
Chemotherapy	0.96	0.93	0.85
Outpatient procedures	0.98	0.89	0.65
Imaging	0.97	0.94	0.72
Radiation therapy	0.99	0.90	0.55
Outpatient physician	0.98	0.95	0.76
Diagnostics	0.97	0.92	0.57
Part B medications	0.94	0.84	0.44
Other Part B	0.92	0.92	0.40
Part D	0.95	1.00	1.00
Total	0.93	0.96	0.71
Total with Part D	0.94	0.97	0.74
**Poor-prognosis cohort**			
Acute hospital	0.95	0.96	0.77
Postacute facility	1.00	0.92	0.74
Hospice	1.00	0.87	0.68
Home health	0.97	0.86	0.58
DME	1.00	0.94	0.20
Chemotherapy	1.00	0.93	0.79
Outpatient procedures	1.00	0.96	0.79
Imaging	0.98	0.96	0.70
Radiation therapy	1.00	0.87	0.51
Outpatient physician	0.97	0.94	0.81
Diagnostics	0.99	0.94	0.71
Part B medications	0.97	0.87	0.48
Other Part B	0.95	0.90	0.38
Part D	0.99	0.97	1.00
Total	0.96	0.95	0.63
Total with Part D	0.96	0.95	0.69

Abbreviation: DME, durable medical equipment.

## Discussion

One method for risk-bearing organizations to succeed is to select efficient practices for their networks or for preferentially directing referrals for patients with cancer. Using national Medicare data, we documented several notable findings that have implications for how ACOs or other risk-bearing organizations might behave in local health care markets. First, acute hospital care and chemotherapy use were the largest drivers of variation within local geographic regions. Moreover, spending in these 2 areas was largely uncorrelated, suggesting that they are not substitutes (though less so in the poor-prognosis cohort). Second, for many areas of the country there is minimal opportunity for risk-bearing organizations to choose among oncology practices. Fully a quarter of HRRs had 3 or fewer practices that met our entry criteria, implying even fewer options in smaller areas. Finally, spending patterns of practices were reasonably correlated across years, suggesting that the spending behavior we identified is at least somewhat enduring, which is necessary if selective referrals is to work as a strategy.

Acute hospital services and provision of chemotherapy were the highest cost areas of spending and also exhibited reasonably high variation. Nonchemotherapy Part B drugs also exhibited high variation. Importantly, total hospital spending was stable to decreased from 2009 to 2017, consistent with prior reports demonstrating decreased spending on surgical care^24^ and hospitalizations for patients undergoing chemotherapy.^7^ In contrast, average spending on chemotherapy increased substantially.^7^ The findings related to the proportion of spending attributed to hospital care were consistent with a prior analysis that examined sources of variation in spending across (rather than within) regions using data ending in 2009.^13^ Since the time of that analysis, however, numerous anticancer therapies have entered the market with very high launch prices, while the prices of many existing anti-cancer therapies have also increased.^25,26,27^ Thus, decisions about chemotherapy use have become an increasingly important driver of variation. Our findings also are consistent with a more recent analysis that examined interregional spending for patients with 5 types of cancer who were undergoing chemotherapy, which found that acute hospital care spending and chemotherapy were the largest contributors to variation across regions.^10^ Nevertheless, the Oncology Care Model evaluation found no effect on use of hospital-based care in its first 3 years, despite this being an area of focus for participating practices.^7^ It also is true that not all spending in oncology treatment is controlled by oncologists (particularly for patients not receiving chemotherapy). For instance, Baumgardner et al^11^ showed that although most spending for conditions such as multiple myeloma, lung, and breast cancer is related to oncology care, 20% to 40% might not be. Thus, the spending patterns we observed may not be fully under the control of oncology practices.

One important tool for risk-bearing organizations is the ability to identify and select lower-spending practices for inclusion in their networks or for targeting referrals. For such a strategy to be effective, there must be variation among practices that does not simply reflect substitution of one type of spending for another, the variation must reflect variation in practice patterns as opposed to nonrandom sorting of patients, and the variation must persist over time. In addition, there must be capacity at lower spending practices to take on more patients. Our findings suggest that such a strategy could be viable in many areas of the country (though not all), with a potential for achieving 10% to 15% overall savings on average. There are other challenges to pursuing such a strategy, however. Network inclusion often is dictated by ownership or other similar affiliations that effectively limit choices for organizations such as hospital-led ACOs. In addition, physicians directing referrals might prefer to keep care within their own system, to enable easier communication with oncologists or information exchange about a patient’s care, such as through a shared electronic health record. Another challenge for hospital-led ACOs is that expensive oncology practices may be a source of profit for hospital systems that is more beneficial to the system than the downstream rewards they might receive from savings.^28^ If the introduction of future alternative payment models from CMS and commercial payers can successfully lower spending, the focus on shifting referral patterns may be less important over time. Finally, HRRs can sometimes be large, and lower-spending practices might not be accessible to all patients in an HRR. The advent of remote monitoring and telehealth strategies over the past several years is 1 potential strategy that could mitigate issues related to travel time.

### Limitations

Our results have limitations. First, though we attempted to identify an incident cohort of patients with newly-diagnosed cancer, some patients might have had recurrent cancers. Second, though we studied a large and comprehensive sample of patients with cancer, we lacked clinical data that could be used for more accurate staging. Thus, there may have been clinical heterogeneity among those at different practices. Indeed, our analyses suggest that there was variation in patient characteristics across practices, suggesting that differential sorting of patients to specific types of practices contributed to the variation we observed. Third, this study included patients in the Medicare program who were enrolled in Part D and may not generalize to other patient populations. Fourth, for risk-bearing organizations to be able to steer patients to low-spending oncology practices, there must be excess capacity in these practices. Our analyses of caseload at the oncologist level, however, suggests that there is capacity. Finally, our analyses focused on spending but did not consider quality, which would be another important consideration for identifying high-value practices.

## Conclusions

In this national cohort study of cancer care for patients in traditional Medicare, our results suggest that there are opportunities for ACOs and other risk-bearing organizations (eg, capitated health plans) to select or drive referrals to lower-spending oncology practices in local markets.

## Notes

Supplement 1. eTable 1. Codes to Identify Patients Admitted With Incident or Poor-Prognosis Cancer eTable 2. Characteristics of Study Population by Cohort eTable 3. Adjusted Correlations Between Spending Categories eAppendix. STROBE Checklist

Supplement 2. Data Sharing Statement
