# Supplementary material for: Opportunities for Savings in Risk Arrangements for Oncologic Care
Source: JAMA Health Forum. 2023 Sep 15;4(9):e233124. doi: 10.1001/jamahealthforum.2023.3124 (PMC10504611; doi:10.1001/jamahealthforum.2023.3124)
Supplement: Supplement 1. — eTable 1. Codes to Identify Patients Admitted With Incident or Poor-Prognosis Cancer eTable 2. Characteristics of Study Population by Cohort eTable 3. Adjusted Correlations Between Spending Categories eAppendix. STROBE Checklist [file jamahealthforum-e233124-s001.pdf]

Supplemental Online Content

Landon BE, Lam MB, Landrum MB, et al. Opportunities for savings in risk arrangements for oncologic care. *JAMA Health Forum*. 2023;4(9):e233124. doi:10.1001/jamahealthforum.2023.3124

eTable 1. Codes to Identify Patients Admitted With Incident or Poor Prognosis Cancer
eTable 2. Characteristics of Study Population by Cohort
eTable 3. Adjusted Correlations Between Spending Categories
eAppendix. STROBE Statement

This supplemental material has been provided by the authors to give readers additional information about their work.

© 2023 Landon BE, et al. *JAMA Health Forum.*

eTable 1: Codes to identify patients admitted with incident or poor prognosis cancer

**Incident**	
ICD-9-CM diagnosis codes<br><br>140.0x-209.36 except 173.xx	ICD-10-CM diagnosis codes<br><br>C00.xx-C96.xx except C44.xx
**Poor Prognosis**	
ICD-9-CM diagnosis codes	ICD-10-CM diagnosis codes
Lung<br>162.0x,162.2x,162.3x,162.4x,162.5x,162.8x,162.9x	Lung<br>C34.xx, C39.xx
Pancreas<br>157.0x,157.1x,157.2x,157.3x,157.8x,157.9x	Pancreas<br>C25.xx
Esophagus<br>150.0x,150.1x,150.2x,150.3x,150.4x,150.5.x,150.8x, 150.9x	Esophagus<br>C15.xx
Stomach<br>151.0x,151.1x,151.2x,151.3x,151.4x,151.5x,151.6x,151.8x, 151.9x	Stomach<br>C16.xx
Meninges<br>192.3x,198.4x,192.1x	Meninges<br>C70.xx
Brain<br>191.0x,191.1x,191.2x,191.3x,191.4x,191.5x,191.6x,191.7x, 191.8x,191.9x	Brain<br>C71.xx
Spinal<br>192.0x	Spinal<br>C72.xx
Liver<br>155.xx	Liver<br>C22.xx
Gallbladder<br>156.xx	Gallbladder<br>C23.xx, C24.xx
Brain Uncertain	Brain Uncertain

© 2023 Landon BE, et al. *JAMA Health Forum.*

237.5x<br>Leukemia<br>205.00, 205.01, 205.02,208.00,204.0x<br>Non-Hodgkin's Lymphoma<br>200.48,200.78,200.65,200.60,202.00,202.70,202.80-202.88,200.2x<br><br>The following cancers require a metastatic code within 30 days:<br><br>CRC<br>153.xx, 154.xx<br>Prostate<br>185.xx<br>Breast<br>174.xx<br>Bladder<br>188.xx<br>Ovary<br>183.xx<br>Kidney<br>189.xx<br>Melanoma<br>172.xx<br>Uterine<br>182.xx<br><br>Metastatic Codes;<br>197.0x,1971.x,197.2x,197.3x.197.4x,197.5x, 197.6x,197.7x,197.8x,198.0x,198.1x,198.2x,198.3x 198.4x,198.5x,198.6x,198.7x,198.81,198.82,198.89	D43.2x<br>Leukemia<br>C91.xx, C92.xx, C93.xx, C95.xx<br>Non-Hodgkin's Lymphoma<br>C83.xx-C86.xx<br><br>The following cancers require a metastatic code within 30 days:<br><br>CRC<br>C18.xx-C20.xx, C26.xx<br>Prostate<br>C61.xx<br>Breast<br>C50.xx<br>Bladder<br>C67.xx, C68.xx<br>Ovary<br>C56.xx, C57.xx<br>Kidney<br>C64.xx, C65.xx<br>Melanoma<br>C43.xx<br>Uterine<br>C54.xx, C55.xx<br><br>Metastatic Codes;<br>C78.xx, C79.xx

© 2023 Landon BE, et al. *JAMA Health Forum.*

© 2023 Landon BE, et al. *JAMA Health Forum.*

eTable 2. Characteristics of Study Population by Cohort

Appendix Table 2: Characteristics of study population by cohort

		Incident (2009-2010)		Incident (2016-2017)		Poor Prognosis (2009-2010)		Poor Prognosis (2016-2017)	
		N=247,801		N=333,320		N=258,710		N=369,389	
		%	Adjusted SD	%	Adjusted SD	%	Adjusted SD	%	Adjusted SD
Age	Mean*	73.8	1.53	74.1	1.29	72.6	1.97	72.9	1.70
	<65	12.1	0.05	9.5	0.04	14.8	0.06	12.1	0.05
	65-69	19.2	0.02	20.8	0.03	22.7	0.03	26.5	0.04
	70-74	22.7	0.02	24.9	0.02	20.1	0.01	21.1	0.02
	75-79	19.3	0.01	20.2	0.01	17.5	0.01	17.6	0.02
	80-84	15.4	0.02	13.5	0.02	14.4	0.03	12.3	0.02
	85+	11.3	0.03	11.1	0.03	10.4	0.03	10.4	0.03
Sex	Male	35.6	0.08	38.3	0.09	45.0	0.05	48.8	0.06
Race	White	82.4	0.14	84.0	0.14	80.9	0.15	82.4	0.14
	Black	9.2	0.11	7.9	0.10	9.9	0.11	8.3	0.10
	Hispanic	5.2	0.07	4.1	0.07	5.7	0.07	4.6	0.07
	Other	3.2	0.07	4.0	0.06	3.5	0.07	4.6	0.06
Medicaid status	Full	25.9	0.13	17.0	0.12	27.0	0.13	18.4	0.13
	Partial	9.7	0.03	7.7	0.04	9.9	0.03	7.9	0.04
	Low-income subsidy	38.8	0.14	26.4	0.14	40.1	0.15	28.1	0.15
Cancer type	Breast	21.2	0.08	21.5	0.08	6.5	0.02	6.2	0.02
	Lung	17.9	0.05	15.6	0.05	30.3	0.06	23.9	0.06
	CRC/intestinal	11.0	0.03	9.2	0.03	6.0	0.01	5.2	0.01
	Lymphoma	7.1	0.02	7.2	0.03	13.2	0.04	10.5	0.03
	Leukemia	4.1	0.02	4.0	0.01	3.8	0.03	10.1	0.04
	Pancreatic	3.2	0.01	3.6	0.02	5.2	0.02	5.6	0.02
	Prostate	2.6	0.04	3.5	0.04	3.6	0.02	4.5	0.04
	Other	32.9	0.14	35.4	0.14	31.4	0.08	34.0	0.09

* age in years

s.d. is Variation in Patient Characteristics Across TINs Net of Sampling Error

© 2023 Landon BE, et al. *JAMA Health Forum.*

eTable 3. Adjusted Correlations Between Spending Categories

Appendix Table 3. Adjusted Correlations Between Spending Categories

Period	Cost category	Incident Cohort					Poor Prognosis Cohort				
		Acute Hospital	Chemo	Outpatient Physician	Imaging	Radiation Therapy	Acute Hospital	Chemo	Outpatient Physician	Imaging	Radiation Therapy
2009 - 2010	Chemotherapy	-0.15					-0.35				
	Outpatient Physician	-0.74	0.55				-0.32	0.96			
	Imaging	-0.77	0.63	0.99			-0.63	0.92	0.94		
	Radiation Therapy	-0.77	0.07	0.86	0.80		-0.92	0.47	0.53	0.76	
	Part B Medications	-0.67	0.40	0.75	0.75	0.62	-0.60	0.54	0.55	0.65	0.77
2016 - 2017	Chemotherapy	-0.22					-0.33				
	Outpatient Physician	-0.78	0.44				-0.12	0.89			
	Imaging	-0.83	0.63	0.94			-0.78	0.80	0.72		
	Radiation Therapy	-0.82	0.17	0.95	0.83		-0.99	0.35	0.20	0.82	
	Part B Medications	-0.55	0.05	0.48	0.53	0.45	-0.53	0.17	0.36	0.59	0.62

© 2023 Landon BE, et al. *JAMA Health Forum.*

STROBE Statement—Checklist of items that should be included in reports of ***cohort studies***

	Item No	Recommendation	Page No
**Title and abstract**	1	(*a*) Indicate the study's design with a commonly used term in the title or the abstract	1
		(*b*) Provide in the abstract an informative and balanced summary of what was done and what was found	2
**Introduction**			
Background/rationale	2	Explain the scientific background and rationale for the investigation being reported	5
Objectives	3	State specific objectives, including any prespecified hypotheses	6
**Methods**			
Study design	4	Present key elements of study design early in the paper	7
Setting	5	Describe the setting, locations, and relevant dates, including periods of recruitment, exposure, follow-up, and data collection	7
Participants	6	(*a*) Give the eligibility criteria, and the sources and methods of selection of participants. Describe methods of follow-up	7-8
		(*b*) For matched studies, give matching criteria and number of exposed and unexposed	NA
Variables	7	Clearly define all outcomes, exposures, predictors, potential confounders, and effect modifiers. Give diagnostic criteria, if applicable	9
Data sources/ measurement	8*	For each variable of interest, give sources of data and details of methods of assessment (measurement). Describe comparability of assessment methods if there is more than one group	7-9
Bias	9	Describe any efforts to address potential sources of bias	NA
Study size	10	Explain how the study size was arrived at	7-8
Quantitative variables	11	Explain how quantitative variables were handled in the analyses. If applicable, describe which groupings were chosen and why	9-10
Statistical methods	12	(*a*) Describe all statistical methods, including those used to control for confounding	10-11
		(*b*) Describe any methods used to examine subgroups and interactions	NA
		(*c*) Explain how missing data were addressed	NA
		(*d*) If applicable, explain how loss to follow-up was addressed	NA
		(*e*) Describe any sensitivity analyses	NA
**Results**			
Participants	13*	(a) Report numbers of individuals at each stage of study—eg numbers potentially eligible, examined for eligibility, confirmed eligible, included in the study, completing follow-up, and analysed	11
		(b) Give reasons for non-participation at each stage	NA
		(c) Consider use of a flow diagram	NA
Descriptive data	14*	(a) Give characteristics of study participants (eg demographic, clinical, social) and information on exposures and potential confounders	11
		(b) Indicate number of participants with missing data for each variable of interest	NA
		(c) Summarise follow-up time (eg, average and total amount)	7-8
Outcome data	15*	Report numbers of outcome events or summary measures over time	11-13

© 2023 Landon BE, et al. *JAMA Health Forum.*

Main results	16	(*a*) Give unadjusted estimates and, if applicable, confounder-adjusted estimates and their precision (eg, 95% confidence interval). Make clear which confounders were adjusted for and why they were included	12
		(*b*) Report category boundaries when continuous variables were categorized	12-13
		(*c*) If relevant, consider translating estimates of relative risk into absolute risk for a meaningful time period	NA
Other analyses	17	Report other analyses done—eg analyses of subgroups and interactions, and sensitivity analyses	NA
**Discussion**			
Key results	18	Summarise key results with reference to study objectives	14
Limitations	19	Discuss limitations of the study, taking into account sources of potential bias or imprecision. Discuss both direction and magnitude of any potential bias	16
Interpretation	20	Give a cautious overall interpretation of results considering objectives, limitations, multiplicity of analyses, results from similar studies, and other relevant evidence	14-16
Generalisability	21	Discuss the generalisability (external validity) of the study results	16
**Other information**			
Funding	22	Give the source of funding and the role of the funders for the present study and, if applicable, for the original study on which the present article is based	disclosure

*Give information separately for exposed and unexposed groups.

Note: An Explanation and Elaboration article discusses each checklist item and gives methodological background and published examples of transparent reporting. The STROBE checklist is best used in conjunction with this article (freely available on the Web sites of PLoS Medicine at http://www.plosmedicine.org/, Annals of Internal Medicine at http://www.annals.org/, and Epidemiology at http://www.epidem.com/). Information on the STROBE Initiative is available at http://www.strobe-statement.org.

© 2023 Landon BE, et al. *JAMA Health Forum.*
