# Supplementary material for: Opportunities for Savings in Risk Arrangements for Oncologic Care
Source: JAMA Health Forum. 2023 Sep 15;4(9):e233124. doi: 10.1001/jamahealthforum.2023.3124 (PMC10504611; doi:10.1001/jamahealthforum.2023.3124)
Supplement: Supplement 2. — Data Sharing Statement [file jamahealthforum-e233124-s002.pdf]

Data Sharing Statement

Landon. Opportunities for Savings in Risk Arrangements for Oncologic Care. *JAMA Health Forum*. Published September 15, 2023. doi:10.1001/jamahealthforum.2023.3124

Data
Data available: No
